# Exogenous hydrogen sulfide restores cardioprotection of ischemic post-conditioning via inhibition of mPTP opening in the aging cardiomyocytes

**DOI:** 10.1186/s13578-015-0035-9

**Published:** 2015-07-30

**Authors:** Hongzhu Li, Chao Zhang, Weiming Sun, Lina Li, Bo Wu, Shuzhi Bai, Hongxia Li, Xin Zhong, Rui Wang, Lingyun Wu, Changqing Xu

**Affiliations:** Department of Pathophysiology, Harbin Medical University, Baojian Road, Harbin, 150081 China; Department of Emergency, Heilongjiang Provincial Hospital, Harbin, 150036 China; The Key Laboratory of Cardiovascular Medicine Research (Harbin Medical University), Ministry of Education, Harbin, 150086 China; Department of Biology, Lakehead University, Thunder Bay, ON P7B 5E1 Canada; Department of Health Science, Lakehead University, Thunder Bay, ON P7B 5E1 Canada

**Keywords:** Hydrogen sulfide, Post-conditioning, Mitochondrial permeability transition pore, Aging cardiomyocytes

## Abstract

The physiological and pathological roles of hydrogen sulfide (H_2_S) in the regulation of cardiovascular functions have been recognized. H_2_S protects against the hypoxia/reoxygenation (H/R)-induced injury and apoptosis of cardiomyocytes, and ischemic post-conditioning (PC) plays an important role in cardioprotection from H/R injury in neonatal cardiomyocytes but not in aging cardiomyocytes. Whether H_2_S is involved in the recovery of PC-induced cardioprotection in aging cardiomyocytes is unclear. In the present study, we found that both H/R and PC decreased cystathionine-γ-lyase (CSE) expression and the production rate of H_2_S. Supplementation of NaHS protected against H/R-induced apoptosis, the expression of cleaved caspase-3 and cleaved caspase-9, the release of cytochrome *c* (Cyt *c*), and mPTP opening. The addition of NaHS also counteracted the reduction of cell viability caused by H/R and increased the phosphorylation of ERK1/2, PI3K, Akt, GSK-3β and mitochondrial membrane potential. Additionally, NaHS increased Bcl-2 expression, promoted PKC-ε translocation to the cell membrane, and activated mitochondrial ATP-sensitive K channels (mitoK_ATP_). PC alone did not provide cardioprotection in H/R-treated aging cardiomyocytes, which was significantly restored by the supplementation of NaHS. In conclusion, our results suggest that exogenous H_2_S restores PC-induced cardioprotection via the inhibition of mPTP opening by the activation of the ERK1/2-GSK-3β, PI3K-Akt-GSK-3β and PKC-ε-mitoK_ATP_ pathways in aging cardiomyocytes. These findings provide a novel target for the treatment of aging ischemic cardiomyopathy.

**Electronic supplementary material:**

The online version of this article (doi:10.1186/s13578-015-0035-9) contains supplementary material, which is available to authorized users.

## Background

Myocardial ischemia (hypoxia)/reperfusion (reoxygenation) causes cardiomyocyte injury, including cardiomyocyte apoptosis and necrosis. Free radical generation, calcium overload and the adhesion of leukocytes are the main mechanisms of ischemia/reperfusion (I/R) injury. In 1986, Murry et al. first reported that ischemic pre-conditioning (IPC) plays an important cardioprotective role against ischemia-induced injuries [1, 2]. However, IPC must be applied before the ischemic event, which is unpredictable, and is impractical in the clinical setting of acute myocardial infarction [3]. According to a similar regimen of brief periods of ischemia applied just after, instead of just before, sustained ischemia was shown to be as protective as preconditioning. In 2003, Zhao et al. [4] first proposed the concept of ischemic post-conditioning (PC). PC is defined as repetitive cycles of briefly interrupted reperfusion applied at the onset of establishing reflow and has been shown to significantly improve outcome following an episode of ischemia [4]. The main theories of PC-induced protection are the preservation of mitochondrial integrity via regulation of the mitochondrial permeability transition pore (mPTP) [2]. Inhibition of mPTP opening is considered to be the final step in a complex series of cellular signalling events preventing cell death [2]. It is thought that PC activates a signal transduction pathway involving the pro-survival kinases phosphatidylinositol-3-OH kinase (P13K) and protein kinase B (Akt) and the p42/p44 extracellular signal-regulated kinases (ERK1/2), and it has been termed the reperfusion injury salvage kinase (RISK) pathway [5–9]. Meanwhile, PC also activates protein kinase C (PKC)-mitochondrial ATP-sensitive potassium (mitoK_ATP_) channels (PKC-mitoK_ATP_ pathway) [5, 9–11]. The RISK and PKC-mitoK_ATP_ pathways terminate with the inhibition of mPTP opening at reperfusion, thus affording cardioprotection in PC [2, 12].

The degeneration, decrease of autophagic capacity and mitochondrial function (mtDNA mutation), increase of oxidative stress and reduced production of endogenous protective substances in ageing cardiomyocytes, leads to a weakening or disappearance of sensitivity of aging cardiomyocytes to PC [13, 14]. Aging hearts are also resistant to the powerful endogenous protections provided by PC. In other words, PC loses its myocardial protective effect in aging hearts [15].

Hydrogen sulfide (H_2_S) is a highly diffusible gasotransmitter that influences cellular and organ functions through a number of different mechanisms [16]. Endogenous production of H_2_S is mainly catalysed by cystathionine β-synthase (CBS), cystathionine-γ-lyase (CSE) and 3-mercaptosulphurtransferase (3-MPST) [17]. CBS and CSE have been consistently shown to produce H_2_S in mammalian tissues, with l-cysteine and/or homocysteine as the main substrate [18, 19]. Recently, 3-MPST, which uses both l-cysteine and alpha ketoglutarate as substrates along with cysteine aminotransferase to produce H_2_S, was identified [20]. Among them, CSE is the major H_2_S-producing enzyme in vascular tissues. The abnormal metabolism and functions of the CSE/H_2_S pathway have been linked to various cardiovascular diseases, including I/R injury, atherosclerosis, hypertension, and oxidative stress [21–26].

It was previously reported that H_2_S is involved in PC-induced cardioprotection [21–26]. Our previous study indicated that exogenous H_2_S recovered cardioprotection from PC in isolated aging rat hearts [27]. However, whether H_2_S plays a key role in the recovery of PC-induced cardioprotection in aging cardiomyocytes is unknown. This study investigated the effect of exogenous H_2_S on the recovery of PC-induced cardioprotection and its possible mechanisms, including the ERK1/2-GSK-3β, PI3K-Akt-GSK-3β and PKC-ε-mitoK_ATP_ pathways and mPTP opening, in aging cardiomyocytes.

## Methods

### Drugs and reagents

Sodium hydrogen sulfide (NaHS), the anti-CSE antibody, PD98059 (an inhibitor of ERK), LY294002 (a PI3K inhibitor), 5-hydroxydecanoate (5-HD, a mK_ATP_ inhibitor), Chelerythrine chloride (Che, a PKC inhibitor) were purchased from Sigma Chemical Co. (St. Louis, MO, USA). The primary antibodies for anti-PKC-ε, anti-cleaved caspase-3 and -9, Bcl-2, cytochrome *c* (Cyt *c*), Na^+^/K^+^-ATPase, cyclin D1, p21^Cip/WAF-1^ and GAPDH were from Santa Cruz (Bergheimer, Germany). Hoechst 33342, JC-1 kit and Calcein-AM were also from Santa Cruz. The anti-ERK1/2 and PI3K-Akt-GSK-3β antibodies were obtained from Cell Signaling Technology (Danvers, USA). Senescence *β*-galactosidase (*β*-gal) staining kit was purchased from Beyotime Institute of Biotechnology (Shanghai, China). Rat advanced glycation end products (AGEs) ELISA kit was purchased from Proteintech Group (Wuhan, China). All other chemicals were from Sigma or Santa Cruz.

### Primary culture of cardiomyocytes

Primary cultures of neonatal cardiomyocytes were prepared as previously described [8, 27]. Newborn Wistar rats, aged 1–3 days and weighing 5–8 g, were used for this study. All animal experiments were conducted in compliance with the Guide for the Care and Use of Laboratory Animals published by the China National Institutes of Health and approved by the Animal Care Committees of Harbin Medical University, China. Briefly, cells were dissociated from minced hearts of 1- to 3-day neonatal Wistar rats with a 0.25% solution of crude trypsin. Cells were cultured as monolayers at a density of 5 × 10^4^ cells/cm^2^ in Dulbecco’s modified Eagle medium (DMEM) equilibrated with humidified air containing 5% CO_2_ at 37°C. The medium contained 10% calf serum and 2 µM fluorodeoxyuridine, the latter to prevent proliferation of non-myocytes.

### Aging of myocardial cells induced by d-galactose

The treatment for d-galactose induction was as previously described [28, 29]. Once the attached cardiomyocytes were beating spontaneously, the DMEM supplemented with 20% fetal calf serum was removed, and DMEM supplemented with different concentrations (0, 0.1, 1, 10, 100 g/L) d-galactose was added to the cardiomyocytes in the culture cluster for a further different incubation period (0, 12, 24, 48, 72 h). The degree of cell aging was observed through SA *β*-Gal Staining and AGEs ELISA Assay. In the present study, we selected 10 g/L d-galactose concentrations for 48 h incubation period.

### Established aging cardiomyocytes model of hypoxia/reoxygenation

A hypoxic condition was produced by D-Hank solution (in mM: 5.37 KCl, 0.44 KH_2_PO_4_, 136.89 NaCl, 4.166 NaHCO_3_, 0.338 Na_2_HPO_4_, 5 d-glucose, pH 7.3–7.4 at 37°C) saturated with 95% N_2_ and 5% CO_2_. The pH was regulated to 6.8 with lactate to mimic ischemic solution. The aging cardiomyocytes were put into a hypoxic incubator that was equilibrated with 1% O_2_/5% CO_2_/94% N_2_. After hypoxia, the culture medium was rapidly replaced with fresh DMEM with 10% fetal bovine serum (normoxic culture solution) for initiating reoxygenation [9].

### Experimental protocols

**Fig. 1:**
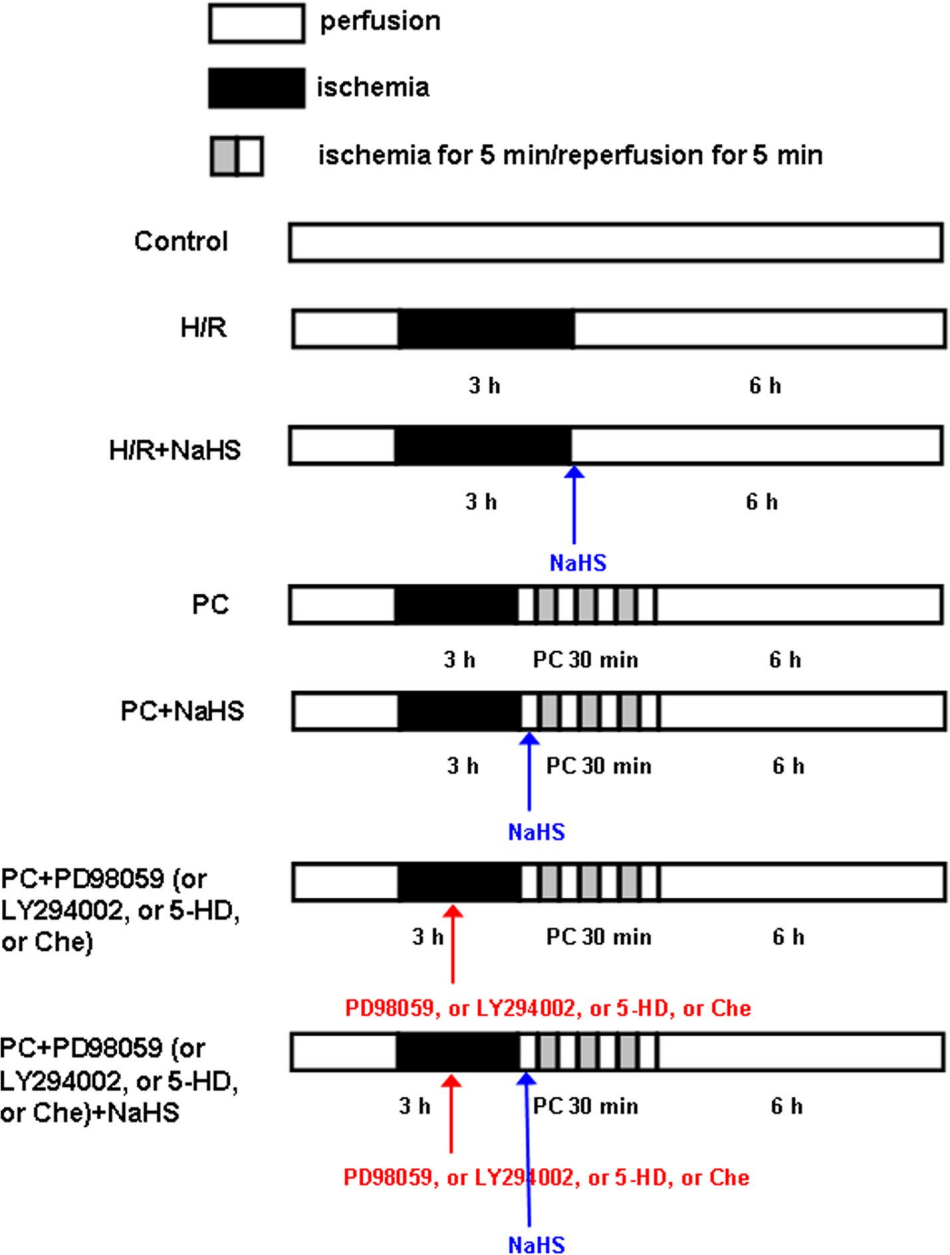
Summary of experimental treatments protocol. The aging cardiomyocytes were exposed to hypoxic culture medium for 3 h and reoxygenated for 6 h by replacing the hypoxic culture medium with fresh DMEM with 10% fetal bovine serum. For details of ischemic postconditioning, NaHS, PD98059, LY294002, 5-HD and Che treatments *see text*.

The aging cardiomyocytes were randomly divided into the following seven groups. Each group included eight samples (n = 8) (Fig. 1): (1) control group (Control): the aging cardiomyocytes were cultured for 9 h with 10% fetal bovine serum-DMEM; (2) hypoxia/reoxygenation group (H/R): the aging cardiomyocytes were exposed to hypoxic culture medium for 3 h and reoxygenated for 6 h by replacing the hypoxic culture medium with fresh DMEM with 10% fetal bovine serum; (3) H/R + H_2_S group: the procedure was similar to that for group 2, except that 100 μM NaHS were added in 6 h reoxygenation; (4) PC group: at the end of 3 h of hypoxia, the aging cardiomyocytes were exposed to normoxic culture solution for 5 min, after which cells were placed in hypoxic solution for 5 min. The PC cycle was repeated three times and followed by 6 h of reoxygenation; (5) PC + H_2_S group: at the end of 3 h of hypoxia, initiated immediately at the onset of reoxygenation, 100 μM NaHS were given at the onset of reoxygenation for 5 min following with 5 min hypoxia. This protocol was repeated for another two times. The cells were then treated as those of group 3; (6) PC + PD98059 (or LY294002, or 5-HD, or Che) group: 10 µM PD98059 (or 10 µM LY294002 or 100 µM 5-HD or 100 µM Che) were added to the medium 40 min before the end of hypoxia. The cells were then treated as those of group 4; (7) PC + PD98059 (or LY294002, or 5-HD, or Che) + H_2_S group: 10 µM PD98059 (or 10 µM LY294002 or 100 µM 5-HD or 100 µM Che) were added to the medium 40 min before the end of hypoxia. The cells were then treated as those of group 5.

The normal cultured cardiomyocytes (without d-galactose-treated cardiomyocytes) were randomly divided into three groups. Each group included eight samples (n = 8): Control group; H/R group; PC group. The cells were treated as those of group 1, 2 and 4, respectively.

### AGEs ELISA Assay

The rat advanced glycation end products (AGEs) assay was performed with AGEs ELISA kit according to the instructions from the manufacturer and was as previously described [29, 30]. The reagents of the test kit were placed at room temperature for 30 min and diluted 1:20 with distilled water. Aliquots of 100 µL of the standards and samples were added to blank micropores and 50 µL enzyme marker solution was added. Microtiter plates were incubated at 37°C for 60 min and then washed five times and put aside for 10–20 s each time. The A and B substrate solutions (50 µL) were added into the microtiter plates for 15 min dark reactions at 37°C. The reaction was terminated by the addition of 50 µL stop solution, and the optical density (OD) at 450 nm was determined by an ultra microplate reader (Bio-Rad Laboratories, Hercules, CA, USA). An AGEs standard curve was generated and the AGEs values of the samples were calculated from the standard curve.

### SA *β*-Gal staining

Senescence-associated *β*-gal (SA *β*-gal) activity was measured with the *β*-gal staining kit at pH 6.0 according to the instructions from the manufacturer [29, 30]. Briefly, the cells were washed in phosphate buffered saline (PBS), fixed for 10–15 min at room temperature with 1 mL of fixative solution and incubated overnight at 37°C with the staining solution mix. Cells were observed for development of the blue coloration with a microscope at a magnification of 400×. Aging cardiomyocytes were assessed by counting the number of cells that displayed blue coloration.

### Observation of cell division index

Briefly, the cells were washed in phosphate buffered saline (PBS) for three times, fixed for 30 min at room temperature with methanol:ice acetic acid (3:1) and incubated for 10 min with Giemsa. Cells were observed with a microscope at a magnification of 400×. Five fields (at least 100 cells of each field) were randomly selected and the percentage of cell division was calculated.

### Detection of cell viability

Cell viability was determined by the 3-(4,5-dimethylthiazol-2-yl)-2,5-diphenyltetrazolium bromide (MTT) assay, as described previously [9, 25, 31]. Cells were cultured in 96-well plates. MTT (final concentration, 0.5 mg/mL) was added to each well under sterile conditions, and the plates were incubated for 4 h at 37°C. The supernatant was removed, and dimethyl sulfoxide (150 μl/well) was added. The plates were then agitated on a plate shaker. The absorbance of each well was measured at 490 nm with a Bio-Rad automated EIA analyser (Bio-Rad Laboratories, Hercules, CA, USA). The viability of control cells was considered 100%, and the others were expressed as percentages of control.

### Determination of H_2_S production

H_2_S production rate was measured as described previously [25]. In brief, after different treatments, the H9C2 cells (cardiomyocytes line) were collected and homogenized in 50 mM ice-cold potassium phosphate buffer (pH 6.8). The flasks containing the reaction mixture (100 mM potassium phosphate buffer, 10 mM l-cysteine, 2 mM pyridoxal 5-phosphate, and 10% cell homogenates) and center wells containing 0.5 mL 1% zinc acetate and a piece of filter paper (2 × 2.5 cm) were flushed with N_2_ gas and incubated at 37°C for 90 min. The reaction was stopped by adding 0.5 mL of 50% trichloroacetic acid, and the flasks were incubated at 37°C for another 60 min. The contents of the center wells were transferred to test tubes, each containing 3.5 mL of water. Then 0.5 mL of 20 mM N, *N*-dimethyl-*p*-phenylenediamine sulfate in 7.2 M HCl and 0.5 mL 30 mM FeCl_3_ in 1.2 M HCl was added. The absorbance of the resulting solution at 670 nm was measured 20 min later with a FLUOstar OPTIMA microplate spectrophotometer.

### Apoptotic rate of cells by flow cytometry assay

The apoptotic rate was measured by flow cytometry as described previously [9, 28, 31]. Cells were washed three times with ice-cold PBS, and then stained with annexin V-fluorescein isothiocyanate for 15 min at room temperature in 200 μl binding buffer. Next, 300 μl binding buffer was added, and the cells were stained with propidium iodide for 30 min at 4°C. The fluorescence of the cells was analyzed by flow cytometry. The percentage of apoptotic cells was determined using Mod Fit LT software (Verity Software House Inc., Topsham, ME, USA).

### Hoechst 33342 staining

Cells were analyzed for apoptosis after visualization of nuclei morphology with fluorescent DNA-binding dye Hoechst 33342, as described previously [9]. After treatment, cells were rinsed with PBS and incubated with 5 µg/mL Hoechst 33342 for 10 min. Nuclei were visualized at 400× magnification using fluorescent microscopy (Nikon Corporation, Tokyo, Japan) at an excitation wavelength of 330–380 nm. Apoptotic nuclei of cells were assessed by counting the number of cells that displayed nuclear morphology changes, such as chromatin condensation and fragmentation.

### Real-time PCR analysis

Total RNA was isolated using an RNeasy Mini Kit (Qiagen, Germantown, MD, USA) and converted to cDNA with an iScriptTM cDNA Synthesis Kit (Bio-Rad, Hercules, CA, USA). Real-time PCR was performed in an iCycler iQ5 apparatus (Bio-Rad) associated with the iCycler optical system software (version 3.1) using SYBR Green PCR Master Mix. The primers of Bcl-2 were 5′-GGCATCTTCTCCTTCCAG-3′ (forward) and 5′-CATCCCAGCCTCCGTTAT-3′ (reverse). Caspase-3 primers were 5′-CAGACAGTGGAACTGACGATGA-3′ (forward) and 5′-AACAGAAACATGCCCCTACCCC-3′ (reverse). Caspase-9 primers were 5′-CCCGTGAAGCAAGGATTT-3′ (forward) and 5′-ACTGTGGGTCTGGGAAGC-3′ (reverse). P-ERK1/2 primers were 5′-ATCCCCCATGGAACGACCTG-3′ (forward) and 5′-ACCCGCCAGGGACAAAAATG-3′ (reverse). The primers of p-PI3K were 5′-CCCTTCTGAACTGGCTTAAAGA-3′ (forward) and 5′-GGACAGTGTAAATTCCTCAATGG-3′ (reverse). The primers for p-Akt were 5′-TGTGACCATGAACGAGTTTGA-3′ (forward) and 5′-GTCGTGGGTCTGGAATGA-3′ (reverse). P-GSK-3β primers were 5′-CGGGACCCAAATGTCAAACA-3′ (forward) and 5′-CGTGACCAGTGTTGCTGAGT-3′ (reverse). The primers of PKC-ε were 5′-CATGGAAGGATAAGCGTTGGT-3′ (forward) and 5′-CCCAAGTCCCGTGTTAAGA-3′ (reverse). The primers for GAPDH were 5′-CTCAACTACATGGTCTACATG-3′ (forward) and 5′-TGGCATGGACTGTGGTCATGAG-3′ (reverse). The cycling conditions were: one cycle of 94°C for 2 min; 30 cycles of 94°C for 30 s, 60°C for 40 s and 72°C for 1 min; and 72°C for 4 min. Relative mRNA quantification was calculated by using the arithmetic formula “2−∆∆CT”, where ∆CT is the difference between the threshold cycle of a given target cDNA and an endogenous reference GAPDH cDNA.

### Detection of Cyt *c* release from mitochondrial

Western blot analysis of Cyt *c* in the cytosolic fraction was performed as described previously [9, 25, 28, 31]. Briefly, cells were harvested, washed twice with ice-cold PBS, and incubated in ice-cold Tris-sucrose buffer (0.35 mM sucrose, 10 mM Tris–HCl at pH 7.5, 1 mM EDTA, 0.5 mM dithiothreitol, 0.1 mM phenylmethylsulphonyl fluoride). After a 40 min incubation, cells were centrifuged at 1,000×*g* for 5 min at 4°C and the supernatant was further centrifuged at 40,000×*g* for 30 min at 4°C. The supernatant was retained as the cytosolic fraction and analyzed by Western blot with a primary rat anti-Cyt *c* monoclonal antibody and a secondary goat anti-rat immunoglobulin G (Promage). GAPDH expression was used as the control.

### Translocation of PKC-ε

Cardiomyocytes were lysed immediately after different treatment by resuspending in lysis buffer [50 mM Tris–HCl, pH 8.0, 150 mM NaCl, 5 mM EDTA, 1% Triton X-100, 0.26% sodium deoxycholate, 50 mM sodium fluoride, 10 mM *β*-glycerophosphate, 0.1 mM sodium orthovanadate, 10 µg/mL leupeptin, and 1 mM phenylmethylsulfonyl fluoride (PMSF)] and incubating on ice for 20 min. Cell debris and insoluble material were cleared by centrifugation at 10,000×*g* for 10 min. The supernatant is called the detergent-solubilized cell lysate. For the preparation of membrane-enriched fractions and subcellular fractions, cells were resuspended in 25 mM Tris–HCl, pH 7.5, 250 mM sucrose, 5 mM MgCl_2_, 100 mM KCl, 10 µg/mL each of aprotinin and leupeptin, and 1 mM PMSF. Cells were disrupted by Dounce homogenization and fractionated by differential velocity centrifugation as previously described [7, 9]. Cell membrane fractions were analyzed by Western blotting with primary rabbit polyclonal isoform specific anti-PKC-ε and secondary goat anti-rat IgG. Na^+^/K^+^-ATPase was used a membrane fractions loading control. The volume of protein bands was quantified using a Bio-Rad Chemi DocTM EQ densitometer and Bio-Rad Quantity One software (Bio-Rad Laboratories, Hercules, USA).

### Measurement of mitochondrial membrane potential (Δψm)

Changes in mitochondrial transmembrane potential (Δψm) were measured with the dye 5,5′,6,6′-tetrachloro-1,1′,3,3′-tetraethylbenzimidazolcarbocyanine iodide (JC-1). Mitochondrial membrane potential was determined as previously described [9]. Briefly, neonatal rat cardiomyocytes (2.5 × 10^5^ cells/60 mm^2^ dishes) were seeded, incubated, and treated for 48 h at 37°C. After experimentation, cells were stained with JC-1 (2 µg/mL, Invitrogen) at 37°C in the dark for 15 min and rinsed three times with cold PBS. Observations were made using a Zeiss LSM 510 inverted confocal scanning microscope. JC-1 monomer (green) fluorescence was observed by excitation with 514 nm and examination of emission at 529 nm. JC-1 aggregate (red) fluorescence was observed by examination of emission at 585/590 nm. At least 100 areas were selected from each image, and the average intensity for each region was quantified. The ratio of JC-1 aggregate to monomer (red/green) intensity for each region was calculated. A decrease in this ratio was interpreted as decrease of Δψm, whereas an increase in this ratio was interpreted as gain in Δψm.

### Assay of mitochondrial permeability transition pore (mPTP) opening

Changes of mitochondrial permeability transition pore (mPTP) opening were measured with coincubation of calcein-AM and cobalt chloride as previously described [9, 32]. Cardiomyocytes were plated in 24-well plates (0.5 × 10^6^ cells/well). After different the treatments, the cells were loaded with calcein-AM 2 µM in presence of 5 mM of cobalt chloride in the dark for for 15 min at 37°C. Fluorescence was measured in a Zeiss LSM 510 inverted confocal scanning microscope at 488 nm excitation and 505 nm emission. Fluorescence intensity of individual cells was measured using SigmaScan Pro 5 software. The fluorescence intensity in control group was considered 100% viable.

### Western blotting analysis

Cells were homogenized in ice-cold lysis buffer containing 50 mM Tris–HCl (pH, 8.0), 150 Mm NaCl, 5 mM EDTA, 1% Triton X-100, 0.26% sodium deoxycholate, 50 mM sodium fluoride, 10 mM *β*-glycerophosphate, 0.1 mM sodium orthovanadate, 10 µg/mL leupeptin, and 50 µg/mL phenylmethylsulfonyl fluoride (PMSF), and incubated on ice for 40 min. The homogenate was centrifuged at 15,000×*g* for 15 min at 4°C to remove cellular debris and isolate total protein. Protein concentrations were determined using a Bradford assay kit (Bio-Rad Laboratories; Hercules, USA). Equal amounts of proteins were boiled and separated with SDS-PAGE and electrophoretically transferred to a nitrocellulose membrane, as described previously [9, 25]. In each lane of a 10% sodium dodecyl sulfate–polyacrylamide gel electrophoresis, equal amounts of proteins were applied, electrophoresed and transferred to a polyvinylidene fluoride membrane. Membranes were blocked with Tris-buffered saline containing 5% non-fat milk at room temperature for 1 h, then incubated overnight at 4°C with primary antibody. The primary antibody dilutions were 1:1,000 for CSE, phosphorylated or total ERK1/2, PI3K, Akt or GSK-3β, and 1:500 for Bcl-2, cleaved caspase-3 and -9, GAPDH, Na^+^-K^+^-ATPase, cyclin D1 and p21^Cip/WAF-1^. The membrane was then washed three times with 1× Tris-buffer saline-Tween 20 (TBST) buffer and incubated in TBST solution with horseradish peroxidase-conjugated secondary antibody (diluted 1:500) for 1 h at room temperature on a shaker. Finally, the membrane was washed with TBST solution for three times. Antibody–antigen complexes were detected using Western Blue Stabilized Substrate for alkaline phosphatase. GAPDH expression was used as the control. The intensities of the protein bands were quantified by a Bio-Rad ChemiDoc™ EQ densitometer and Bio-Rad Quantity One software (Bio-Rad Laboratories).

### Statistical analysis

All data were expressed as the mean ± SE and represented at least three independent experiments. Statistical comparisons were made using student’s *t* test or one-way ANOVA followed by a post hoc analysis (Tukey test) where applicable. Significance level was set at p < 0.05.

## Results

### PC plays cardioprotection in primary cultured neonatal cardiomyocytes

To observe the protective role of PC on the H/R-induced injury in primary cultured neonatal cardiomyocytes, we detected cell viability, cell apoptosis, mRNA level of caspase-3, caspase-9 and Bcl-2. Our data showed that H/R decreased cell viability (p = 0.0003), increased the percentage of apoptotic cells (p = 0.0008) and mRNA level of caspase-3 (p = 0.0006), caspase-9 (p = 0.0001) and Bcl-2 (p = 0.0002) compared with control group (p < 0.05). Compared with H/R group, PC increased cell viability (p = 0.0005) and Bcl-2 mRNA level (p = 0.0007), decreased the percentage of apoptotic cells (p = 0.0007) and mRNA level of caspase-3 (p = 0.0003) and caspase-9 (p = 0.0009) (p < 0.05), suggesting that PC decreased apoptosis by H/R (Additional file 1: Figures S1, S2).

### d-Galactose induces aging in cardiomyocytes

To investigate whether d-galactose successfully induces the aging phenotype in cardiomyocytes, we examined the AGE content, which is considered to be an important marker of cardiac aging. Our results showed that incubation of cardiomyocytes with 10 g/L d-galactose for 48 h significantly increased the AGE content compared with other all groups. Cell viability was not changed by treating the cells with 10 g/L d-galactose for 48 h (Table 1).

**Table 1:** The AGEs content and cell viability of different concentrations of d-galactose for different times

	AGEs content (pg/mL)	Cell viability (%)
d-Galactose concentration (g/L)^a^		
0	88 ± 13	100 ± 23
0.1	191 ± 22*	92 ± 12
1	389 ± 90*	93 ± 34
10	924 ± 93*	95 ± 18
100	230 ± 45*	93 ± 26
Time (h)^b^		
0	95 ± 12	100 ± 13
12	448 ± 82*	94 ± 21
24	577 ± 75*	91 ± 19
48	939 ± 79*	96 ± 25
72	668 ± 102*	93 ± 18

^a^The AGEs content and cell viability of different concentrations of d-galactose for 48 h. * p < 0.05 vs. 0 g/L d-galactose group.

^b^The AGEs content and cell viability of 10 g/L d-galactose for different times. All data were from eight independent experiments. * p < 0.05 vs. 0 h group.

**Fig. 2:**
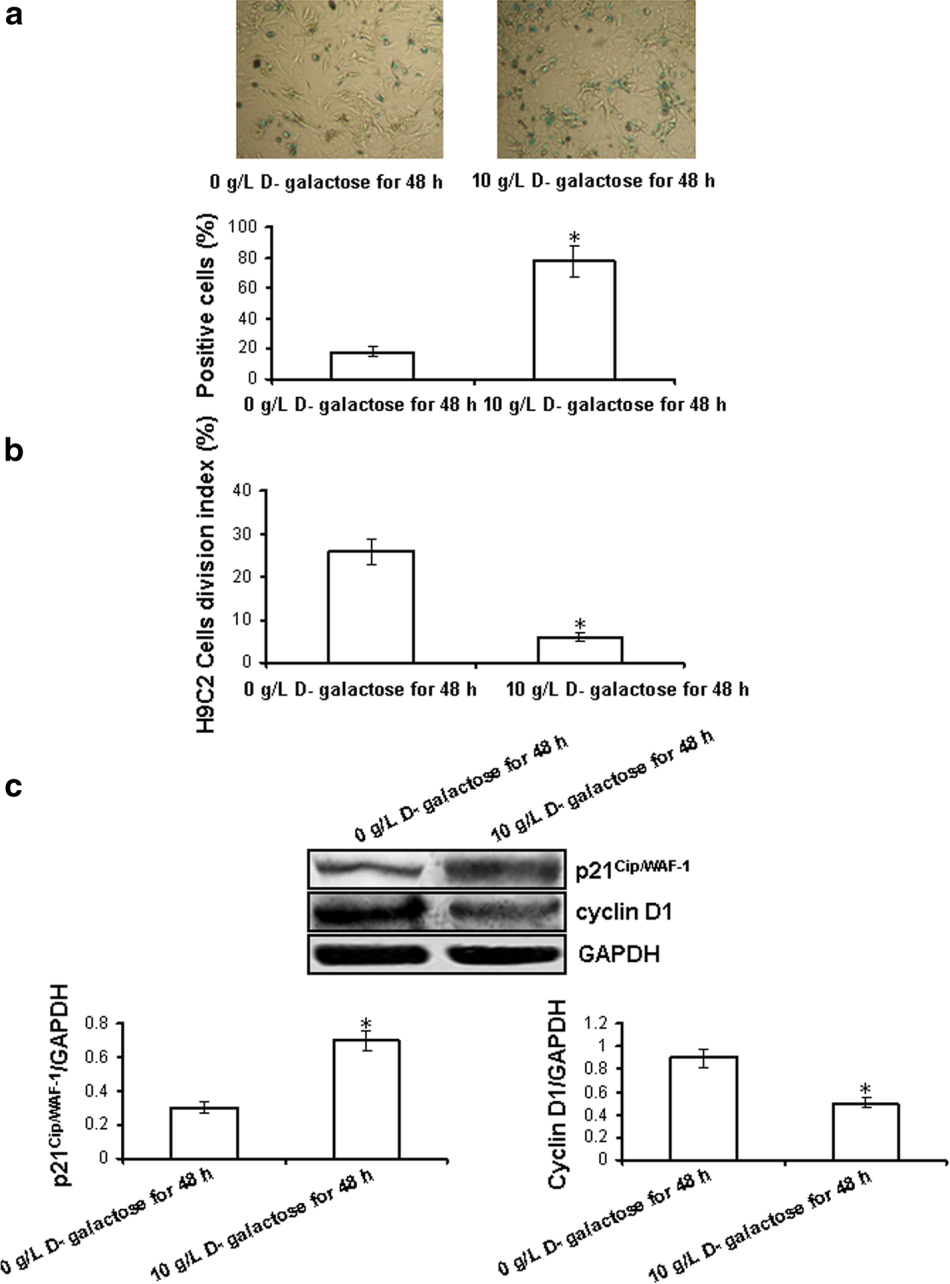
Response of neonatal rat cardiomyocytes to 10 g/L d-galactose for 48 h. **a** The number of SA *β*-gal-positive cardiomyocytes. Phase-contrast images showing morphologic changes and stained cells. (SA-*β*-gal positive cells; *Blue*, magnification ×400). The aging cardiomyocytes in at least five random fields were counted. **b** H9C2 cells division index. Five fields (at least 100 cells of each field) were randomly selected and the percentage of cell division was calculated. **c** The expression of p21^Cip/WAF-1^ and cyclin D1. The intensity of each band was quantified by densitometry, and data were normalized to the GAPDH signal. All data were from four independent experiments. *p < 0.05 vs. 0 g/L d-galactose for 48 h group.

To further confirm this phenomenon, we performed SA *β*-Gal staining and evaluated the H9C2 cell division index (primary cultured neonatal cardiomyocytes do not divide) and the expression of cyclin D1 and p21^Cip/WAF-1^. We found that the number of SA *β*-gal-positive cardiomyocytes and the expression of p21^Cip/WAF-1^ were significantly increased while the H9C2 cell division index and cyclin D1 expression were markedly decreased after incubation with 10 g/L d-galactose for 48 h (p < 0.05 vs 0 g/L d-galactose for 48 h group) (Fig. 2).

### H_2_S production rate and CSE expression changes using the d-galactose age-induced cardiomyocytes

**Fig. 3:**
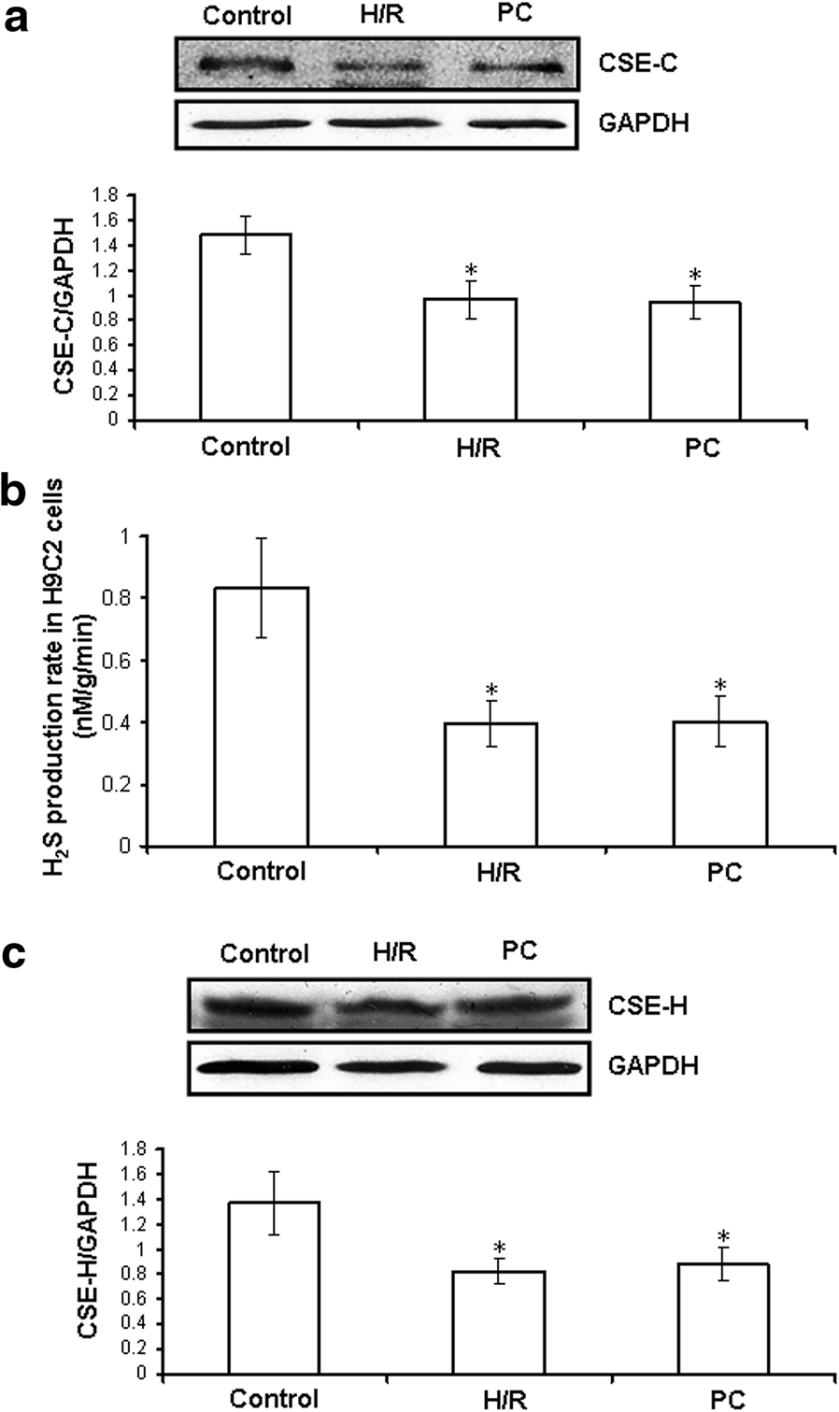
H_2_S production rate and CSE expressions changes using the d-galactose age-induced cardiomyocytes. **a** The expression of CSE protein (CSE-C) in primary cultures of neonatal cardiomyocytes. H_2_S production rate (**b**) and CSE (CSE-H) expressions (**c**) in H9C2 cells. The intensity of each band was quantified by densitometry, and data were normalized to the GAPDH signal. All data were from four independent experiments. *p < 0.05 vs. control group.

CSE is a H_2_S-producing key enzyme in the cardiovascular system. In order to detect whether the loss of PC cardioprotection in the aging cardiomyocytes may be related with the decrease of endogenous H_2_S, we examined H_2_S production rate and CSE expression changes using d-galactose age-induced cardiomyocytes and H9C2 cells. Compared with the control group, the H_2_S production rate (p = 0.0004) and CSE expression (CSE-C: p = 0.006; CSE-H: p = 0.004) (CSE-C, primary cultures of neonatal cardiomyocytes; CSE-H, H9C2 cells) were significantly decreased in both the H/R and PC groups (p < 0.05). The difference in the H_2_S production rate and CSE expression between the H/R and PC groups was not significant (Fig. 3).

### Exogenous H_2_S increases cell viability and decreases apoptosis in the d-galactose age-induced primary cultured neonatal cardiomyocytes

**Fig. 4:**
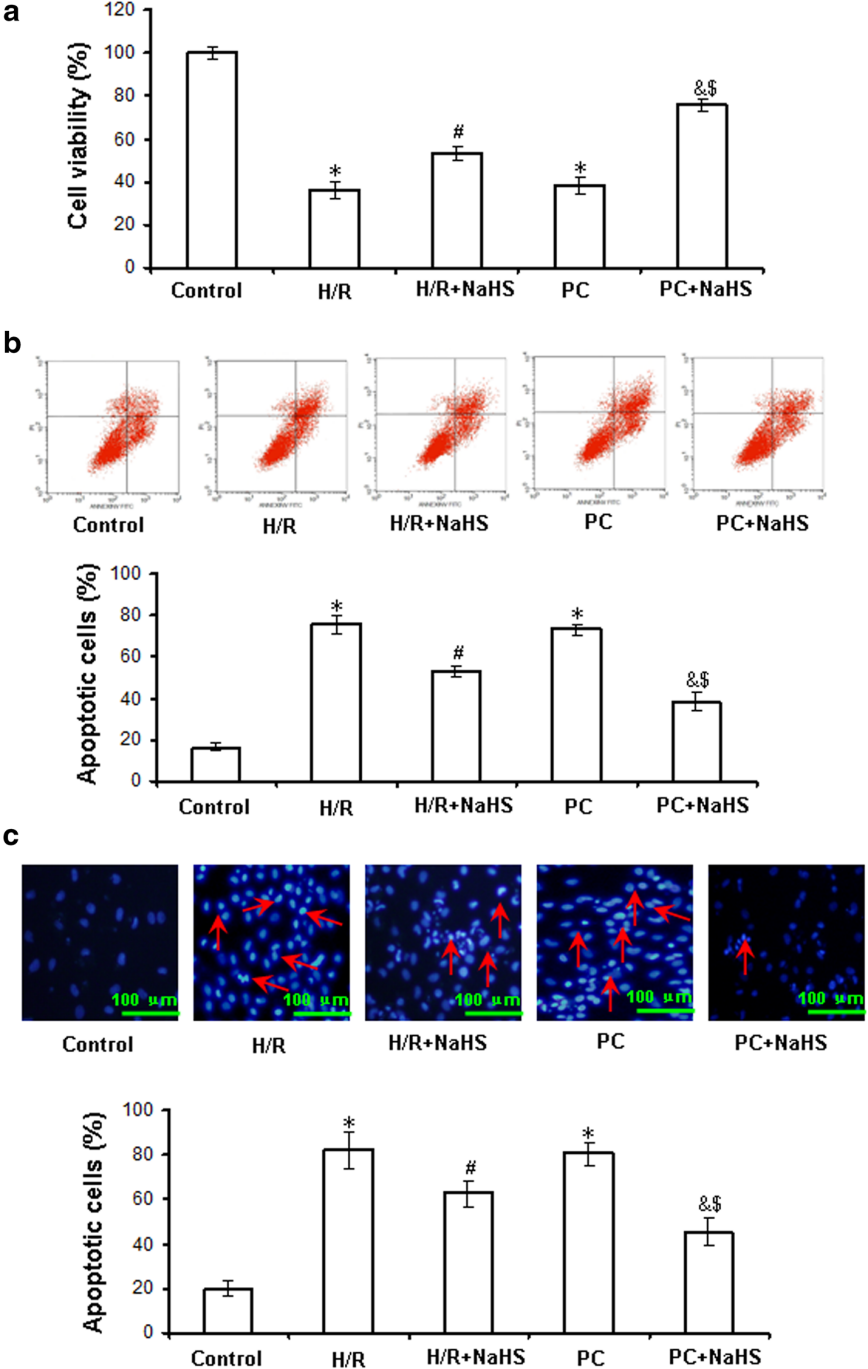
The effect of exogenous H_2_S on the cell viability and apoptosis in the d-galactose age-induced primary cultured neonatal cardiomyocytes. **a** Cell viability was measured by MTT assay. The cells incubated with control medium were considered 100% viable. **b** Apoptosis analyzed by flow cytometry. **c** Detection of nuclear morphology in apoptotic cells by Hoechst 33342 staining. Apoptotic cells were identified as cells with condensed, disrupted nuclei (*arrow*, Hoechst staining, ×400). *Scale bar* 100 μm. Apoptotic cells in at least five random fields were counted. All data were from four independent experiments. *p < 0.05 vs. control group; ^#^p < 0.05 vs. H/R group; ^&^p < 0.05 vs. PC group; ^$^p < 0.05 vs. H/R + NaHS group.

To assay whether exogenous H_2_S restores PC-induced cardioprotection, we observed cell viability through MTT mothed in the d-galactose age-induced cardiomyocytes. Cell viability was markedly reduced in the H/R group and could be reversed by NaHS co-treatment. The cell viability of the PC group was similar to the H/R group. Compared with the H/R + NaHS and PC groups, cell viability was obviously increased in the PC + NaHS group (p = 0.003, p < 0.05), indicating that exogenous H_2_S restored the PC-induced increase in cell viability (Fig. 4a).

To investigate whether exogenous H_2_S protects against PC-induced cardiac apoptosis, we examined cardiomyocyte apoptosis by flow cytometry. H/R significantly increased the percentage of apoptotic cells (p = 0.0003, p < 0.05 vs. control group). Compared with the H/R group, H/R + NaHS reduced the apoptotic rate (p = 0.0006, p < 0.05). The apoptotic rate of the PC group was similar to the H/R group. The percentage of apoptotic cells in the PC + NaHS group was significantly decreased compared with the H/R + NaHS and PC groups (p = 0.005, p < 0.05) (Fig. 4b). We further looked for the morphological changes in the nuclei of cardiomyocytes with Hoechst 33342. The number of apoptotic nuclei showing the typical features of fragmentation and condensation in the H/R group was significantly increased compared to the control group (p = 0.0003, p < 0.05). The number of apoptotic nuclei was significantly decreased in the H/R + NaHS group compared with that in the H/R group (p = 0.0002, p < 0.05). The number of apoptotic nuclei in the PC group was similar to the H/R group. Compared with the H/R + NaHS and PC groups, the number of apoptotic nuclei was significantly decreased in the PC + NaHS group (p = 0.009, p < 0.05), suggesting that exogenous H_2_S prevented PC-inhibited apoptosis by H/R (Fig. 4c).

### Exogenous H_2_S inhibits pro-apoptotic factors and promotes anti-apoptotic factors in the d-galactose age-induced primary cultured neonatal cardiomyocytes

H/R damages mitochondria, then the release of pro-apoptotic factors (cleaved caspase-3, cleaved caspase-9 and Cyt *c*) from injured mitochondria is increased, which finally lead to apoptosis. To further indicate exogenous H_2_S restores PC-induced cardioprotection via inhibition of apoptosis, we examined the expression of cleaved caspase-3, cleaved caspase-9 and Bcl-2 in the whole cell lysates as well as Cyt *c* in the cytosolic fraction by western blot in the d-galactose age-induced cardiomyocytes.

**Fig. 5:**
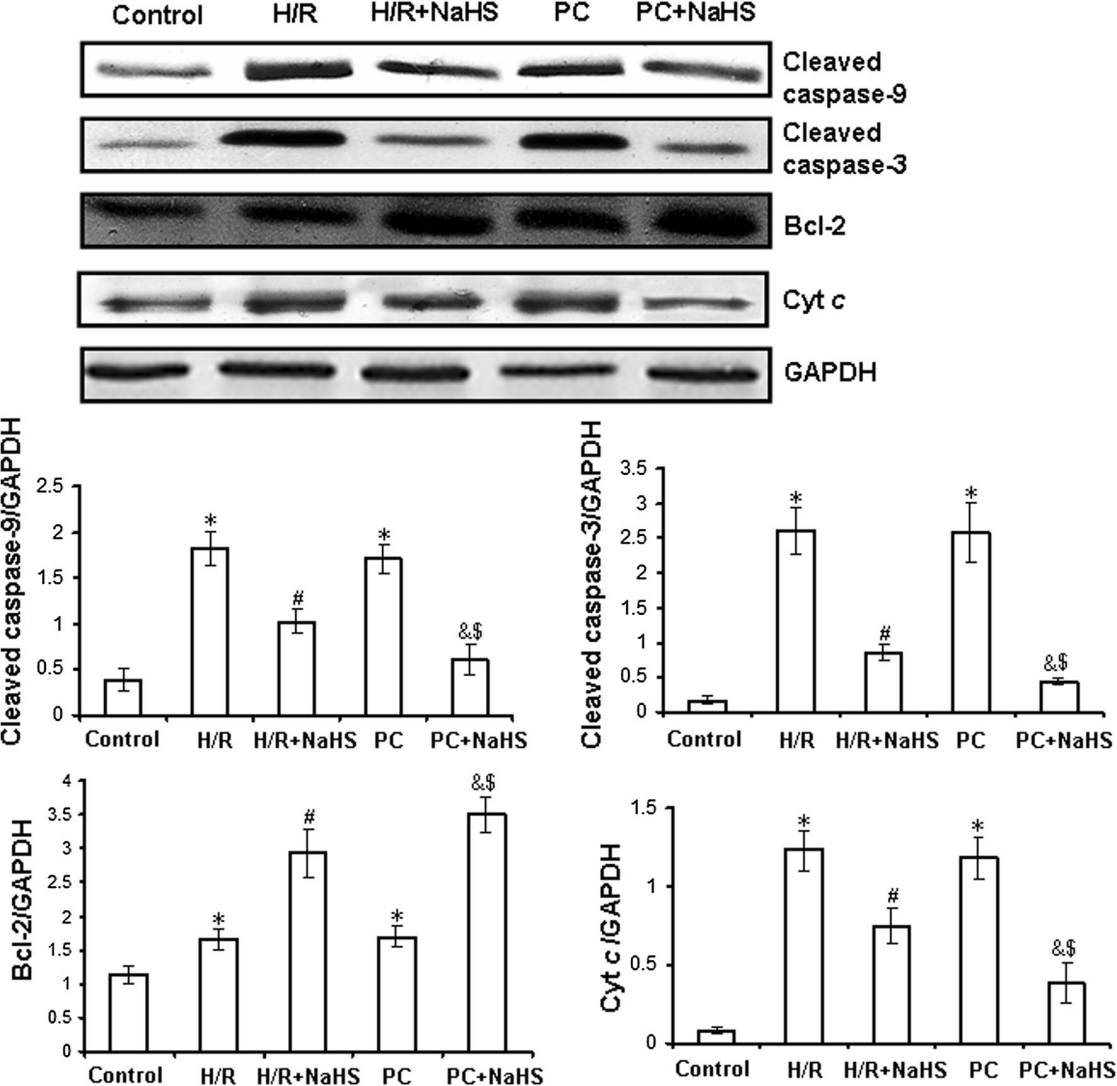
Expression of Bcl-2, cleaved caspase-3 and cleaved caspase-9, cytosolic Cyt *c* in the d-galactose age-induced primary cultured neonatal cardiomyocytes by western blot analysis. The intensity of each band was quantified by densitometry, and data were normalized to the GAPDH signal. All data were from four independent experiments. *p < 0.05 vs. control group; ^#^p < 0.05 vs. H/R group; ^&^p < 0.05 vs. PC group; ^$^p < 0.05 vs. H/R + NaHS group.

Figure 5 shows that the expression of cleaved caspase-3 (p = 0.0009), cleaved caspase-9 (p = 0.0007) and Cyt *c* (p = 0.0005) and Bcl-2 (p = 0.0004) was increased in the H/R group compared with the control group (p < 0.05). Compared with H/R, H/R + NaHS showed decreased expression of cleaved caspase-3 (p = 0.004), cleaved caspase-9 (p = 0.009) and Cyt *c* (p = 0.008) but increased expression of Bcl-2 (p = 0.0007) (p < 0.05). The results of the PC group were similar to those of the H/R group. PC + NaHS treatment markedly decreased the expression of cleaved caspase-3 (p = 0.004), cleaved caspase-9 (p = 0.009) and Cyt *c* (p = 0.0008) and increased the expression of Bcl-2 (p = 0.0007) in comparison with the H/R + NaHS and PC groups (p < 0.05). These results suggest that exogenous H_2_S restores the anti-apoptotic effect of PC against H/R by preventing the mitochondrial apoptotic pathway (Cyt *c*–caspase-9–caspase-3).

### Exogenous H_2_S up-regulates ERK1/2-GSK-3β and PI3K-Akt-GSK-3β pathways in the d-galactose age-induced primary cultured neonatal cardiomyocytes

ERK1/2-GSK-3β and PI3K-Akt-GSK-3β pathways play an important role in the cellular metabolism and gene expression related to growth and apoptosis. To detect whether exogenous H_2_S restores PC protective role by activating ERK1/2-GSK-3β and PI3K-Akt-GSK-3β pathways, we observed the mRNA level and activity of phosphorylated ERK1/2, GSK-3β, PI3K and Akt in the d-galactose age-induced cardiomyocytes.

**Fig. 6:**
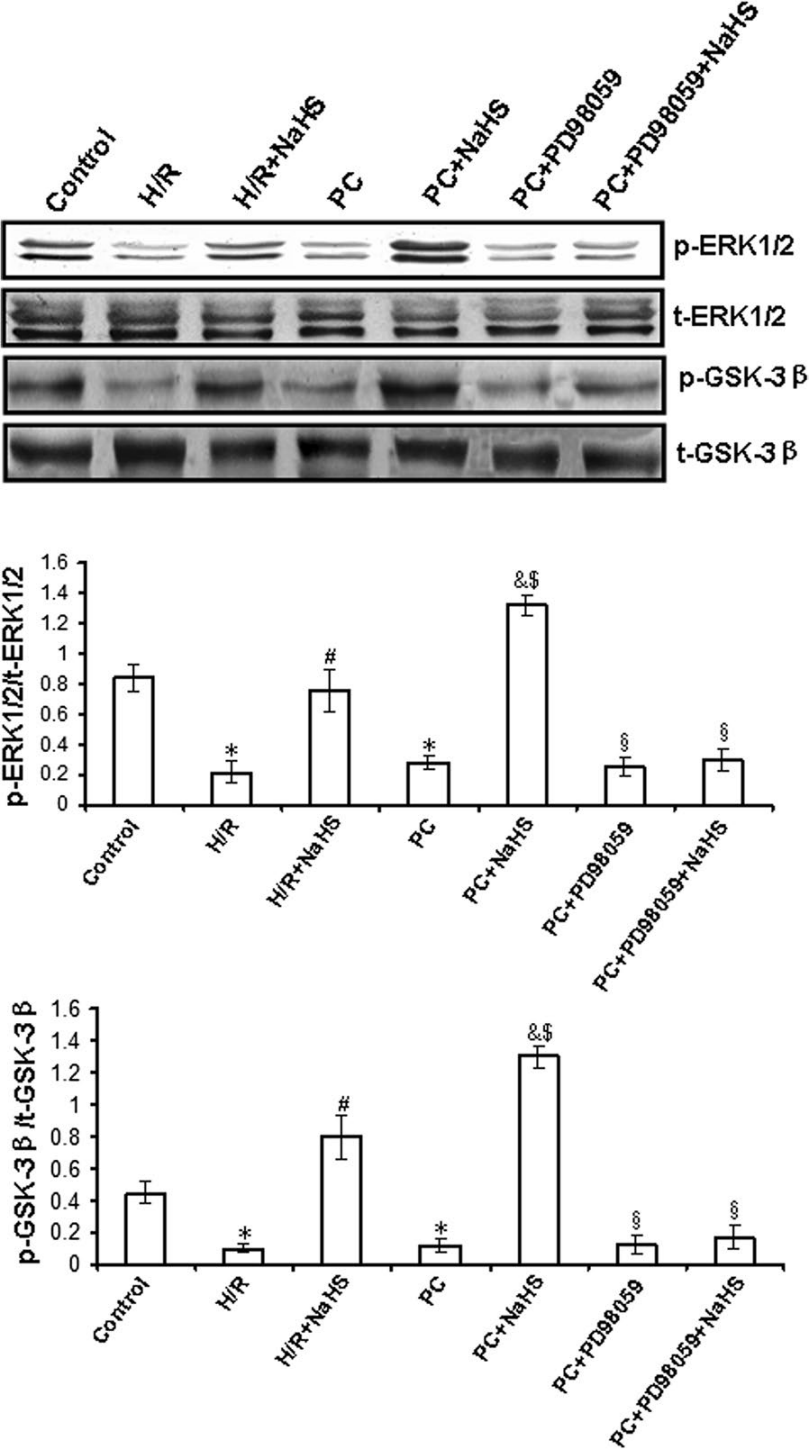
The change of ERK1/2 and GSK-3β activities in the d-galactose age-induced primary cultured neonatal cardiomyocytes. The different cells were collected and subjected to western blot. The *graphs* represent the optical density of the band of phosphorylated ERK1/2 and GSK-3β normalized with that of total ERK1/2 and GSK-3β, respectively. All data were from four independent experiments. *p < 0.05 vs. control group; ^#^p < 0.05 vs. H/R group; ^&^p < 0.05 vs. PC group; ^$^p < 0.05 vs. H/R + NaHS group; ^§^p < 0.05 vs. PC + NaHS group.

The activity and the mRNA level of phosphorylated ERK1/2 (p = 0.0005) and GSK-3β (p = 0.0003) in the H/R group was significantly lower than that in the control group (p < 0.05). The activity and the mRNA level of phosphorylated ERK1/2 (p = 0.0009) and GSK-3β (p = 0.0004) was significantly higher in the H/R + NaHS group than in the H/R group (p < 0.05). The changes in phosphorylated ERK1/2 and GSK-3β activities and mRNA levels in the PC group were similar to those in the H/R group. In the PC + NaHS group, the activity and the mRNA level of phosphorylated ERK1/2 (p = 0.004) and GSK-3β (p = 0.006) was increased to a larger extent than that in the H/R + NaHS and PC groups (p < 0.05), but an ERK1/2 inhibitor (PD98059) abolished these effects of NaHS. The total amount of ERK1/2 and GSK-3β protein remained unchanged with the different stimulations. These data demonstrate that exogenous H_2_S restores the cardioprotection of PC through the activation of the ERK1/2-GSK-3β pathway (Fig. 6; Additional file 1: Figure S3).

**Fig. 7:**
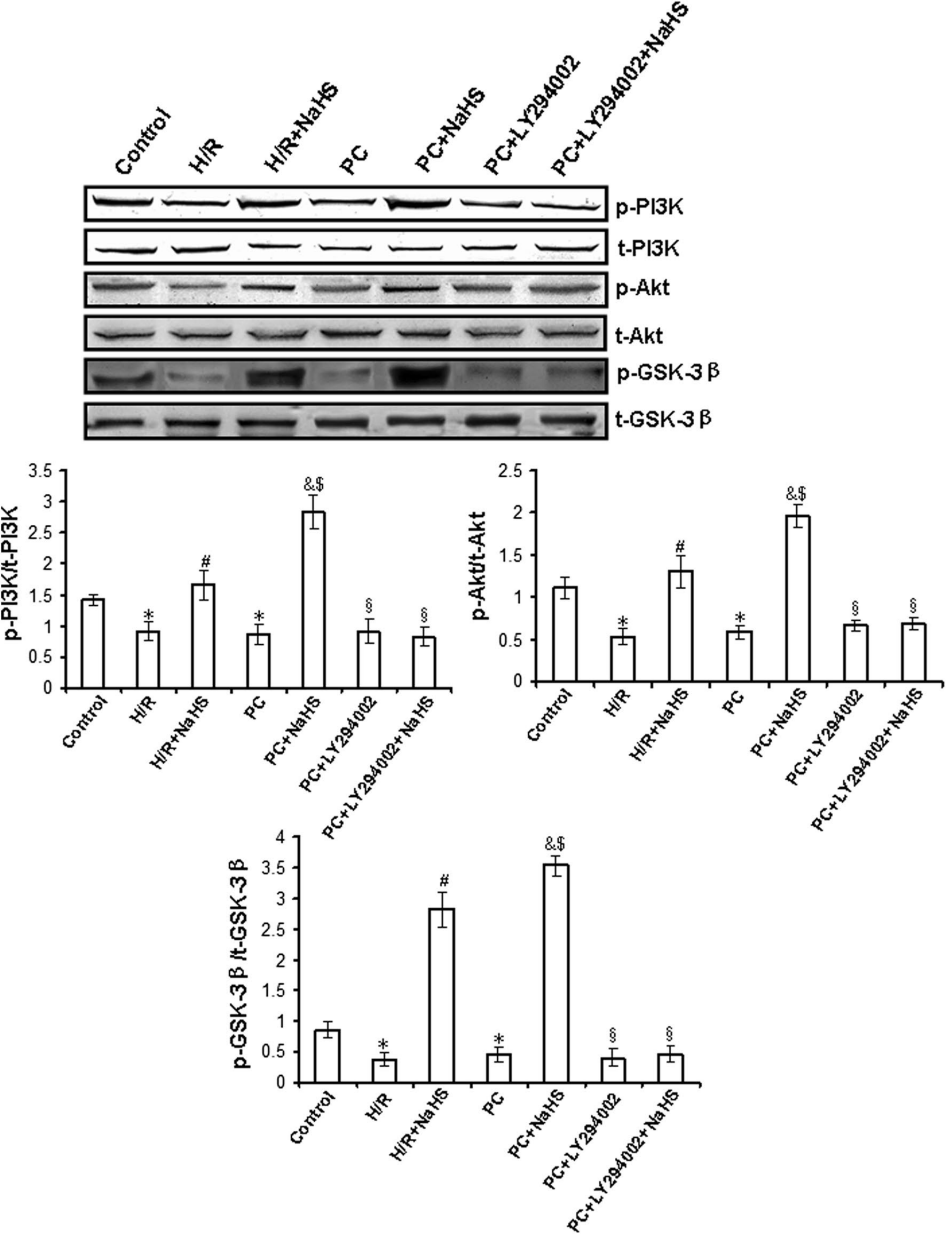
The change of PI3K, Akt and GSK-3β activities in the d-galactose age-induced primary cultured neonatal cardiomyocytes. The different cells were collected and subjected to western blot. The *graphs* represent the optical density of the band of phosphorylated PI3K, Akt and GSK-3β normalized with that of total PI3K, Akt and GSK-3β, respectively. All data were from four independent experiments. *p < 0.05 vs. control group; ^#^p < 0.05 vs. H/R group; ^&^p < 0.05 vs. PC group; ^$^p < 0.05 vs. H/R + NaHS group; ^§^p < 0.05 vs. PC + NaHS group.

Figure 7 and Additional file 1: Figure S4 showed that the activity and the mRNA level of phosphorylated PI3K (p = 0.0003), Akt (p = 0.0005) and GSK-3β (p = 0.0009) were decreased in the H/R group compared with those in the control group (p < 0.05). The activity and the mRNA level of PI3K (p = 0.0008), Akt (p = 0.0004) and GSK-3β (p = 0.0006) was increased in the H/R + NaHS group compared with that in the H/R group (p < 0.05). Their changes in the PC group were similar to the H/R group. PC + NaHS significantly increased the activity and the mRNA level of PI3K (p = 0.008), Akt (p = 0.003) and GSK-3β (p = 0.007) in comparison with the H/R + NaHS and PC groups (p < 0.05), and an inhibitor of PI3K (LY294002) significantly suppressed the NaHS-induced phosphorylation of PI3K, Akt and GSK-3β. The total amount of PI3K, Akt and GSK-3β protein remained unchanged with different stimulations. These results suggest that exogenous H_2_S restored the protective effects of PC by the up-regulation of the PI3K-Akt-GSK-3β pathway.

### Exogenous H_2_S promotes translocation of PKC-ε to the cell membrane in the d-galactose age-induced primary cultured neonatal cardiomyocytes

**Fig. 8:**
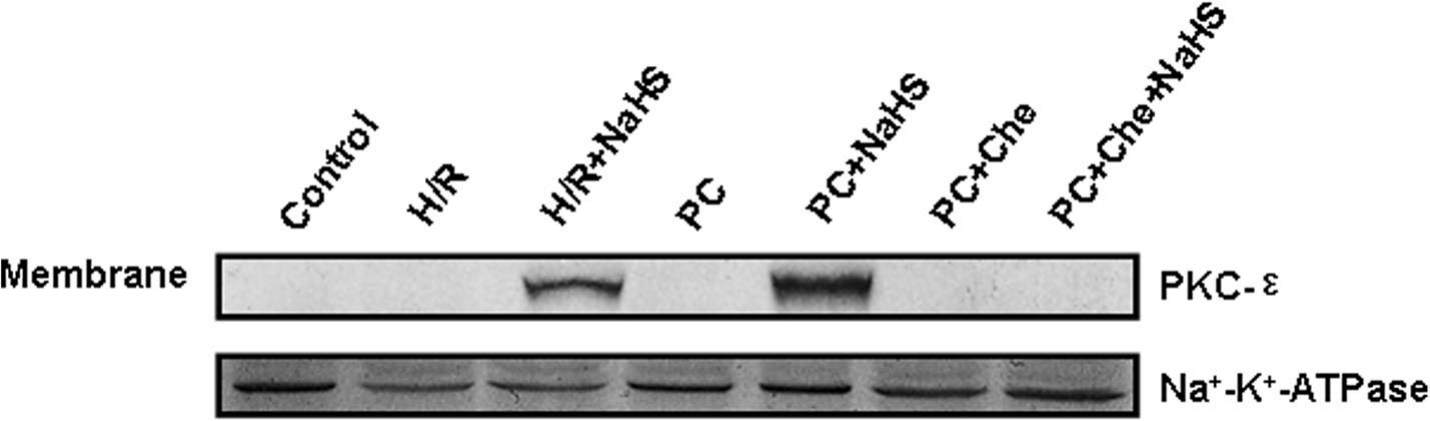
Translocation of PKC-ε to cell membrane by Western blot in the d-galactose age-induced primary cultured neonatal cardiomyocytes. Control, H/R, PC, PC + Che and PC + Che + NaHS groups showed no translocation of PKC-ε to cell membrane. H/R + NaHS and PC + NaHS groups showed translocation of PKC-ε to cell membrane. Na^+^/K^+^-ATPase was used a membrane fraction loading control.

The PKC-ε activates the mK_ATP_ and then inhibits mPTP opening, which finally plays protection during PC. In this study, we examined the translocation of PKC-ε in the cell membrane. Our results demonstrated that the novel PKC-ε isoform is activated by NaHS. Figure 8 and Additional file 1: Figure S5 showed that PKC-ε translocated to the cell membrane in the H/R + NaHS and PC + NaHS groups but not in the Control, H/R, H/R + Che (a specific inhibitor of PKC), H/R + Che + NaHS, PC, PC + Che and PC + Che + NaHS groups.

### Exogenous H_2_S increases mitochondrial membrane potential (Δψm) and inhibits mPTP opening in the d-galactose age-induced primary cultured neonatal cardiomyocytes

**Fig. 9:**
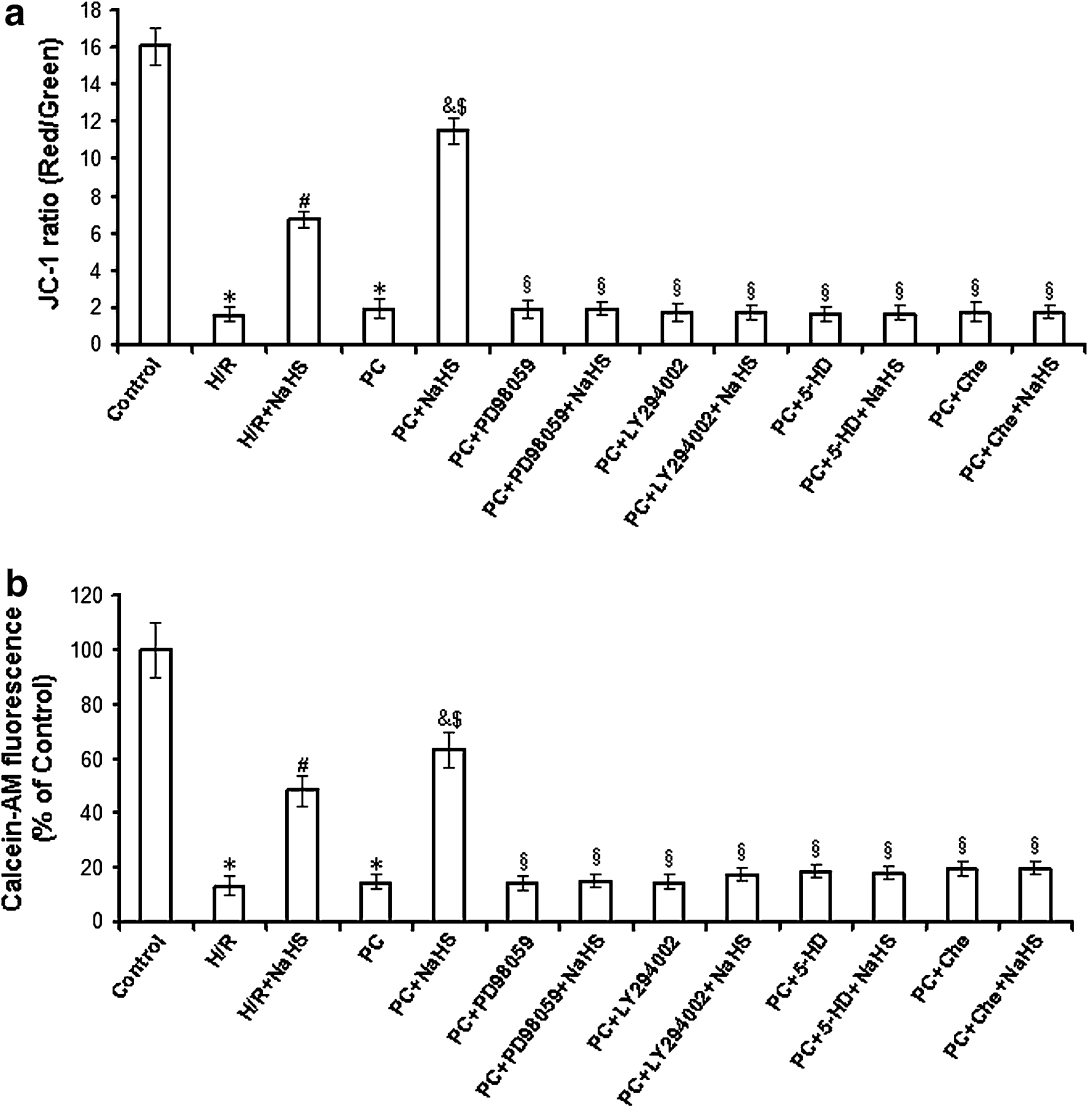
Analysis of the mitochondrial membrane potential (Δψm) and mPTP opening in the d-galactose age-induced primary cultured neonatal cardiomyocytes. **a** JC-1 measured the mitochondrial membrane potential (Δψm). The cells of different groups were stained with JC-1 probe and imaged by fluorescent microscope. JC-1 spontaneously forms J-aggregate and exhibits red fluorescence under a high potential. The JC-1 monomeric form shows green fluorescence under a low potential. The individual red and green average fluorescence intensities are expressed as the ratio of red to green fluorescence. **b** Changes of mPTP opening were measured with calcein-AM. The fluorescence intensity in control group were considered 100% viable. The changes of fluorescence intensity are inversely proportional with degree of the mPTP opening. All data were from four independent experiments. *p < 0.05 vs. control group; ^#^p < 0.05 vs. H/R group; ^&^p < 0.05 vs. PC group; ^$^p < 0.05 vs. H/R + NaHS group; ^§^p < 0.05 vs. PC + NaHS group.

The decreased Δψm induces mPTP opening and then damages celles. To explore the effect of exogenous H_2_S on the recovery of PC-induced cardioprotection, we observed the changes of Δψm and mPTP. As shown in Fig. 9a, the Δψm in the H/R group was significantly decreased (vs. the control group, p = 0.0003, p < 0.05). The Δψm in the H/R + NaHS group was significantly increased (vs. the H/R group, p = 0.0008, p < 0.05). The Δψm of the PC group was similar to that in the H/R group. The Δψm in the PC + NaHS group was higher than that of the H/R + NaHS and PC groups (p = 0.0009, p < 0.05). PD98059, LY294002, 5-HD (an inhibitor of mK_ATP_) and Che reversed the increase of the Δψm by NaHS.

At the same time, we assessed mPTP opening with calcein-AM in the different groups. As shown in Fig. 9b, the fluorescence intensity was reduced in the H/R group (p = 0.0007, p < 0.05 vs. control group). H/R + NaHS resulted in increased fluorescence intensity as compared to the H/R group (p = 0.0006, p < 0.05). The fluorescence intensity of the PC group was similar to the H/R group. PC + NaHS further increased the fluorescence as compared to the H/R + NaHS and PC groups (p = 0.0008, p < 0.05), while PD98059, LY294002, 5-HD and Che blocked the increase in fluorescence intensity caused by PC + NaHS.

## Discussion

The senescent phenotype is associated with a typical gene expression profile and higher SA *β*-gal activity [28, 33]. At same time, AGE content is also considered to be one of the most important markers in aging [34]. Cell cycle progression is determined by the formation of protein complexes between cyclins and cyclin-dependent kinases (cdk), and cyclin D associates with cdk2, cdk4, and cdk6 for the determination of early G1 progression [35]. Also involved in the control of cyclin–cdk activity in all phases of the cell cycle is p21^Cip/WAF-1^ [35]. The increase of cyclin D1 and the decrease of p21^Cip/WAF-1^ reflect cell growth and division. It is well known that the cell division index reflects cell growth, and senescent cells cannot undergo cell division. Our data showed that the number of SA *β*-gal-positive cells, the AGE content and the expression of p21^Cip/WAF-1^ were increased and cyclin D1 expression was decreased in cardiomyocytes, while the H9C2 cell division index was markedly decreased, in treated with 10 g/L d-galactose for 48 h (Table 1; Fig. 1). This indicates the successful establishment of the d-galactose-induced cardiomyocyte aging model used in the present study. As primary cultured neonatal cardiomyocytes cannot divide, the cell division index was assessed using H9C2 cells (cardiomyocytes line).

I/R can induce tissue and organ injury, as well as apoptosis. The mechanisms of apoptosis include the activation of mitochondrial death receptors and/or endoplasmic reticulum stress pathways [9]. The mitochondrial pathway is the major apoptosis-inducing pathway. The intrinsic or mitochondrial pathway transduces a wide spectrum of death signals that originate from both outside and inside the cells. The stimulation of these pathways triggers the translocation of death-promoting proteins (e.g., Cyt *c*) from the mitochondria to the cytoplasm [36, 37] and the activation of a class of cysteine proteases called caspases, which initiate apoptosis [37]. PC protects cardiac against I/R injury and apoptosis by improving cardiac function, decreasing infarct size and inhibiting apoptosis, among other mechanisms [5, 7, 38]. However, it was recently reported that PC loses its myocardial protective effect in ageing hearts [13–15]. The main reason is that, during ageing, cardiomyocytes undergo complex changes, which finally result in loss of contractile function and loss of endogenous protection against irreversible injury [13–15]. Aging affects cardiomyocytes at several subcellular and molecular levels, including alterations at the level of the DNA, increased oxidative stress, changes in gene/protein expression and posttranslational modifications, and the handling of cellular ‘waste’ material by autophagy [13–15]. All these alterations decrease the tolerance of cardiomyocytes to stress [13–15]. Our data showed that in the normal primary cultured neonatal cardiomyocytes, compared with H/R group, PC increased cell viability and Bcl-2 mRNA level, decreased the percentage of apoptotic cells and mRNA level of caspase-3 and caspase-9 (Additional file 1: Figures S1, S2). In the d-galactose age-induced cardiomyocytes, the difference in cell viability, the apoptotic rate, and cleaved caspase-9, cleaved caspase-3, Bcl-2 and Cyt *c* expression between the H/R and PC groups in aging cardiomyocytes was not significant (Figs. 3, 4). These results indicate that PC plays protective role in the normal primary cultured neonatal cardiomyocytes but no in the aging cardiomyocytes.

H_2_S has long been considered as a pungent cytotoxic gas but now is regarded as the third endogenous signalling gasotransmitter. H_2_S is generated in mammalian tissues from homocysteine and cysteine in a reaction catalysed by CBS and CSE, the key enzymes of the transulphuration pathway of methionine metabolism [17–19, 37]. CBS is mainly expressed in the brain and peripheral nervous system, whereas CSE is mostly found in vascular and nonvascular smooth muscle cells [17–19, 37]. Yang and colleagues [24] generated mice lacking CSE (CSE knockout) that had much lower H_2_S concentrations in the aorta and heart, and their findings suggested that CSE is the major enzyme that maintains H_2_S concentrations in the cardiovascular system. Recent studies have shown that increased endogenous CSE/H_2_S pathway expression or exogenous H_2_S is involved in PC-induced cardioprotection [39]. Therefore, we proposed that the loss of cardioprotection from PC in ageing cardiomyocytes is associated with changes in the CSE/H_2_S pathway. In the present study, we found that compared with the control group, the H_2_S production rate of H9C2 cells and CSE expression in ageing cardiomyocytes and H9C2 cells were significantly decreased in both the H/R and PC groups. The difference in the H_2_S production rate and CSE expression between the H/R and PC groups was not significant (Fig. 2). We then used NaHS solution as a source of H_2_S and observed that compared with the H/R group, cell viability and Bcl-2 expression were significantly increased, while the apoptotic rate and expression of cleaved caspase-9, cleaved caspase-3 and Cyt *c* were obviously decreased in the H/R + NaHS group, demonstrating that exogenous H_2_S reduced H/R-induced cardiomyocyte injury. This is consistent with previous reports [23, 24]. Meanwhile, we also found that PC + NaHS further enhanced the cardioprotective roles of H/R + NaHS. These data suggest that exogenous H_2_S restored PC-induced cardioprotective effects in ageing cardiomyocytes.

To further explore the mechanisms by which exogenous H_2_S restored PC-induced cardioprotective effects, we assessed changes in mitochondrial membrane potential, mPTP opening and relevant signalling pathways. ERK1/2 is a member of the mammalian mitogen-activated protein kinase family (MAPK), which has a unique role in the regulation of cellular metabolism and gene expression (such as GSK-3β) related to growth and apoptosis [9, 39, 40]. The PI3K-Akt pathway is proximal to endothelial nitric oxide synthase [9, 41, 42], and its activation also results in the phosphorylation of downstream GSK-3β and, consequently, regulating cell biological and pathological functions. The current study suggests that the up-regulation of the ERK1/2-GSK-3β and PI3-Akt-GSK-3β pathways may contribute to the protective effect of PC through the inhibition of mPTP opening [43, 44]. In addition, PKC-ε activates mitoK_ATP_ and inhibits mPTP opening, which is involved in cardioprotection during PC [45]. We showed here that H/R + NaHS promoted the phosphorylation of the ERK1/2-GSK-3β and PI3K-Akt-GSK-3β pathways and the translocation of PKC-ε to the cell membrane, increased the mitochondrial membrane potential and inhibited mPTP opening (Figs. 5, 6, 7, 8; Additional file 1: Figures S3, S4, S5). PC + NaHS further enhanced the effects of H/R + NaHS. PD98059 (an inhibitor of ERK1/2 signalling), LY 294002 (a PI3K inhibitor), Che (a PKC inhibitor) and 5-HD (a mitoK_ATP_ inhibitor) abolished the effects of PC + NaHS, respectively (Figs. 5, 6, 7, 8; Additional file 1: Figures S3, S4, S5). Taken together, these findings suggest that exogenous H_2_S plays an important role in the recovery of PC-induced cardioprotection by inhibiting mPTP opening through the phosphorylation of the ERK1/2-GSK-3β and PI3K-Akt-GSK-3β pathways, as well as the activation of the PKC-ε-mitoK_ATP_ pathway.

**Fig. 10:**
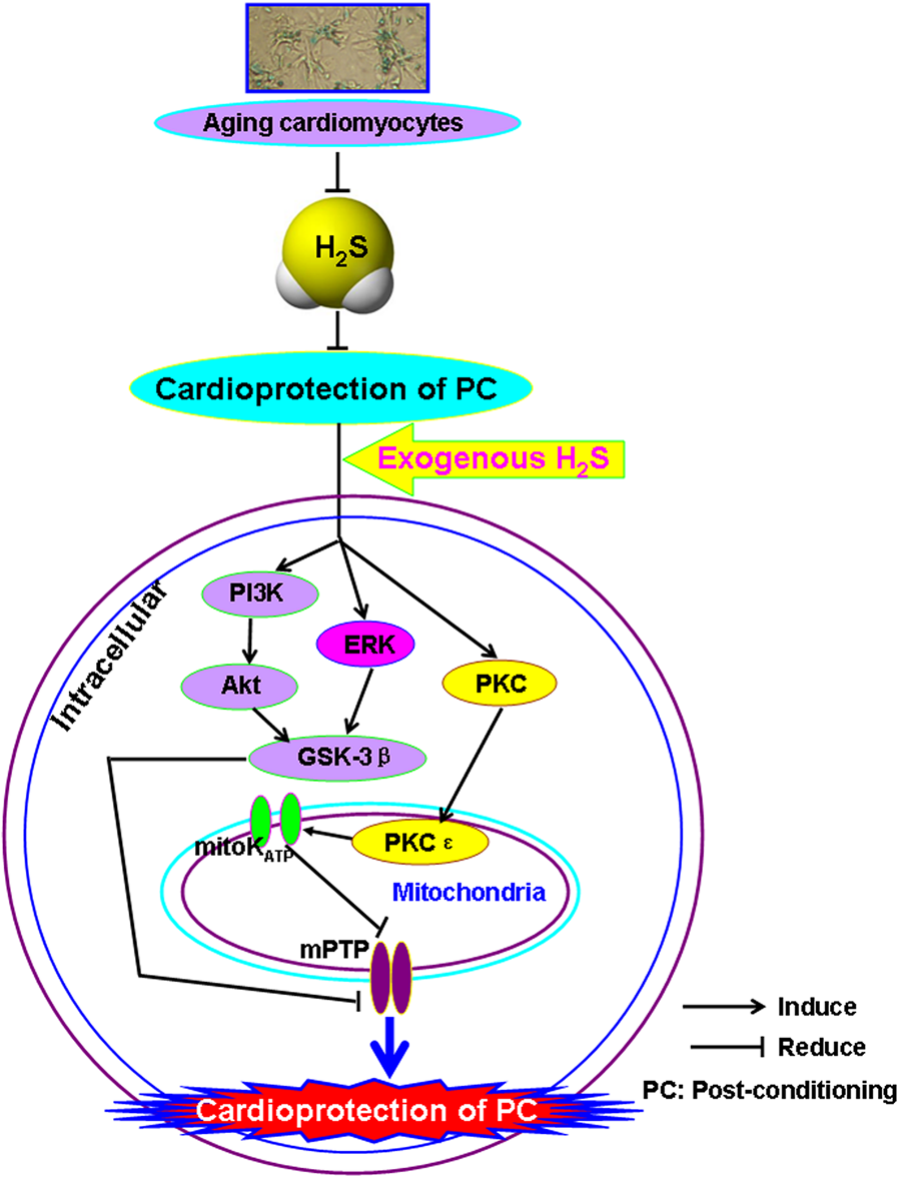
Involvement of exogenous H_2_S in recovery of PC-induced cardioprotection in aging cardiomyocytes. Exogenous H_2_S mediates recovery of PC-induced cardioprotection via inhibition of mPTP opening by up-regulation of ERK1/2-GSK-3β, PI3K-Akt-GSK-3β and PKC-mK_ATP_ pathways.

In summary (Fig. 10), the present research demonstrated that (1) H/R causes injury to aging cardiomyocytes and that exogenous H_2_S reduces H/R injury. (2) In aging cardiomyocytes, PC loses its cardioprotective effects on H/R injury and exogenous H_2_S recovers PC-induced cardioprotection by inhibiting mPTP opening via the activation of the ERK1/2-GSK-3β, PI3K-Akt-GSK-3β and PKC-ε-mK_ATP_ pathways. Dissection of the mechanisms underlying H_2_S protection should facilitate novel preventive and therapeutic approaches for ischemic cardiomyopathy in the aging process.

## Additional file

Additional file 1:
**Figure S1.** The change of the cell viability and apoptosis in the normal primary cultured neonatal cardiomyocytes. **Figure S2.** The change of caspase-3, caspase-9 and Bcl-2 mRNA levels in the normal primary cultured neonatal cardiomyocytes. **Figure S3.** The effect of exogenous H_2_S on p-ERK1/2 and p-GSK-3β mRNA levels in the d-galactose age-induced cardiomyocytes. **Figure S4.** The effect of exogenous H_2_S on p-PI3K, p-Akt and p-GSK-3β mRNA levels in the d-galactose age-induced cardiomyocytes. **Figure S5.** The effect of exogenous H_2_S on PKC-ε mRNA levels in the cell membrane in the d-galactose age-induced cardiomyocytes.
