# Supplementary material for: Exogenous hydrogen sulfide restores cardioprotection of ischemic post-conditioning via inhibition of mPTP opening in the aging cardiomyocytes
Source: Cell Biosci. 2015 Jul 30;5:43. doi: 10.1186/s13578-015-0035-9 (PMC4520088; doi:10.1186/s13578-015-0035-9)
Supplement: Additional file 1: — Figure S1. The change of the cell viability and apoptosis in the normal primary cultured neonatal cardiomyocytes. Figure S2. The change of caspase-3, caspase-9 and Bcl-2 mRNA levels in the normal primary cultured neonatal cardiomyocytes. Figure S3. The effect of exogenous H2S on p-ERK1/2 and p-GSK-3β mRNA levels in the d-galactose age-induced cardiomyocytes. Figure S4. The effect of exogenous H2S on p-PI3K, p-Akt and p-GSK-3β mRNA levels in the d-galactose age-induced cardiomyocytes. Figure S5. The effect of exogenous H2S on PKC-ε mRNA levels in the cell membrane in the d-galactose age-induced cardiomyocytes. [file 13578_2015_35_MOESM1_ESM.doc]

**Additional file 1**

**Exogenous hydrogen sulfide restores cardioprotection of ischemic post-conditioning via inhibition of mPTP opening in the aging cardiomyocytes**

Hongzhu Li, Chao Zhang, Wei ming Sun, Lina Li, Bo Wu,

Shuzhi Bai, Hongxia Li, Xin Zhong, Rui Wang, Lingyun Wu, Changqing Xu

**Additional results**

**
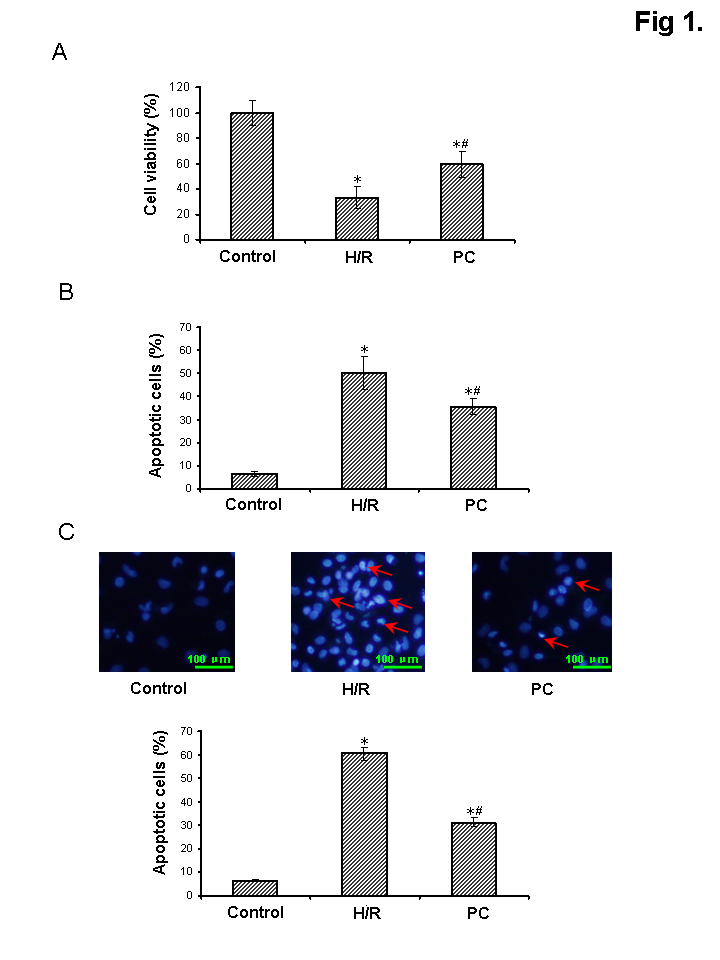
**

**Figure S1. The change of the cell viability and apoptosis in the normal primary cultured neonatal cardiomyocytes.** A. Cell viability was measured by MTT assay. The cells incubated with control medium were considered 100% viable. B. Apoptosis analyzed by flow cytometry. C. Detection of nuclear morphology in apoptotic cells by Hoechst 33342 staining. Apoptotic cells were identified as cells with condensed, disrupted nuclei (arrow, Hoechst staining,×400). Scale bar =100 μm. Apoptotic cells in at least five random fields were counted. All Data were from four independent experiments. * p<0.05 *vs*. control group; # p<0.05 *vs*. H/R group.


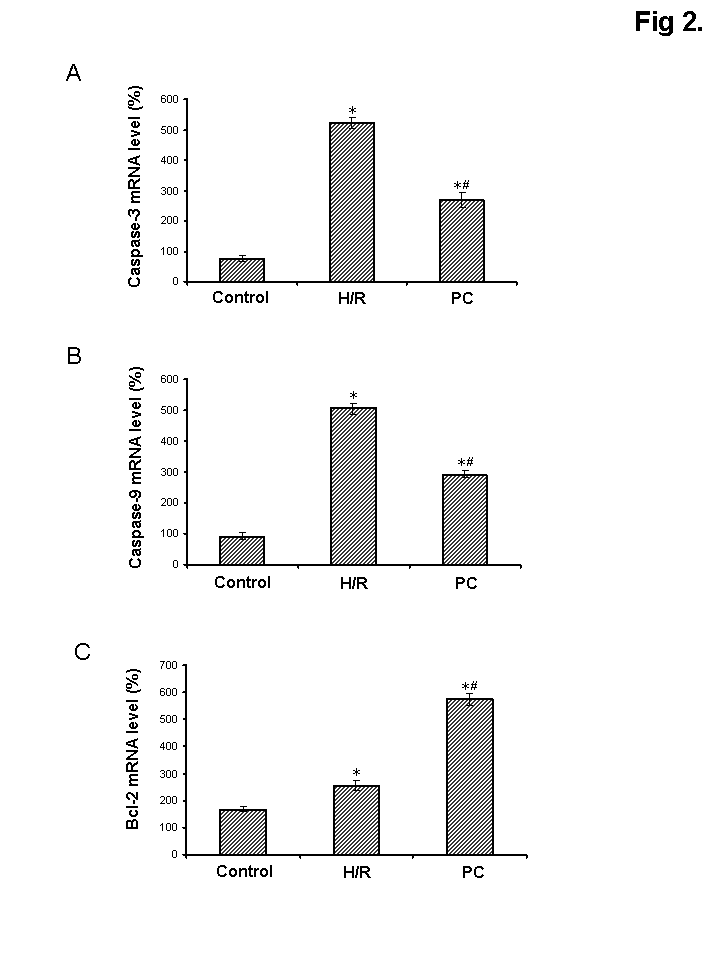


**Figure S2. The change of caspase-3, caspase-9 and Bcl-2 mRNA levels in the normal primary cultured neonatal cardiomyocytes.** The level of caspase-3 (A), caspase-9 (B) and Bcl-2 (C) mRNA was tested using Real-Time PCR. The data were normalized to the GAPDH. All data were from four independent experiments. * p<0.05 *vs*. control group; # p<0.05 *vs*. H/R group.


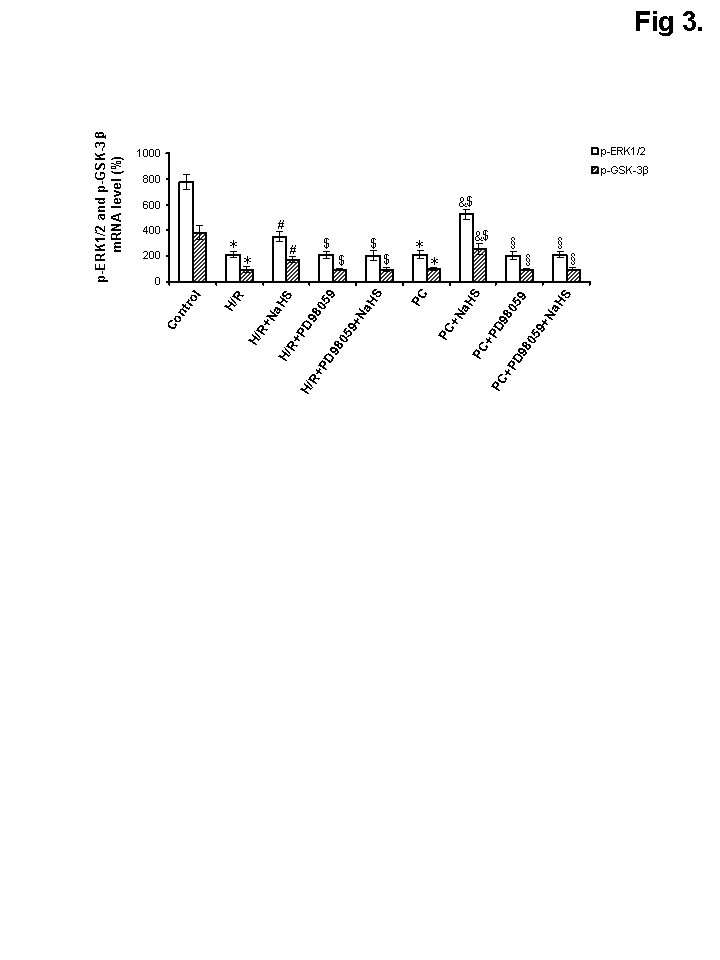


**Figure S3. The effect of exogenous H_2_S on p-ERK1/2 and p-GSK-3β mRNA levels in the D-galactose age-induced cardiomyocytes.** The level of p-ERK1/2 and p-GSK-3β mRNA was tested using Real-Time PCR. The data were normalized to the GAPDH. All data were from four independent experiments. * p<0.05 *vs*. control group; # p<0.05 *vs*. H/R group; & p<0.05 *vs*. PC group; $ p<0.05 *vs*. H/R + NaHS group; § p<0.05 *vs*. PC + NaHS group.


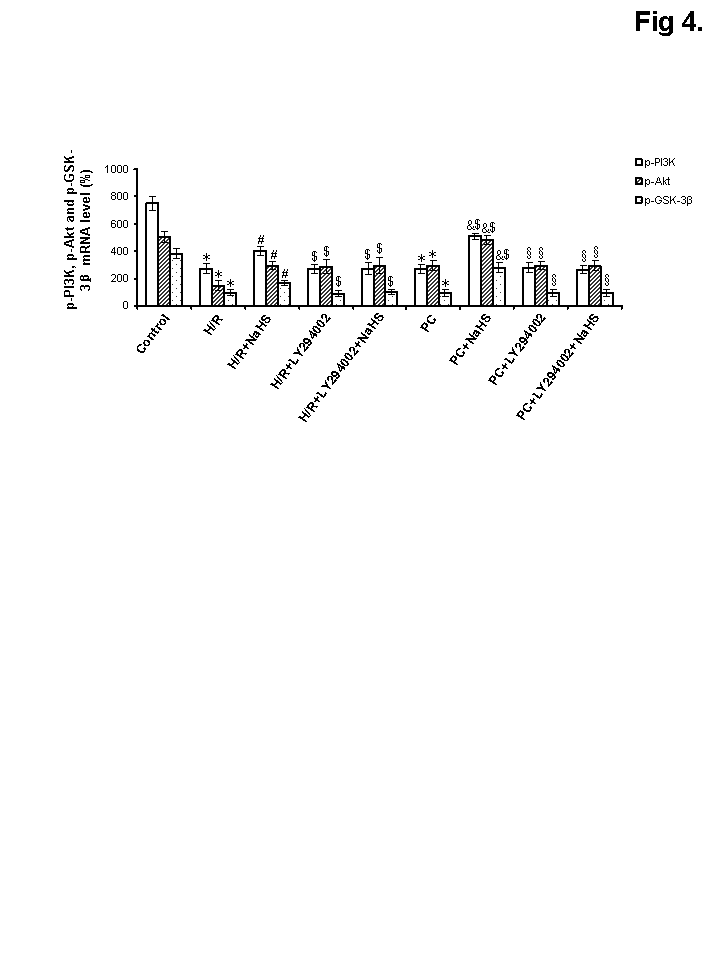


**Figure S4. The effect of exogenous H_2_S on p-PI3K, p-Akt and p-GSK-3β mRNA levels in the D-galactose age-induced cardiomyocytes.** The level of p-PI3K, p-Akt and p-GSK-3β mRNA was tested using Real-Time PCR. The data were normalized to the GAPDH. All data were from four independent experiments. * p<0.05 *vs*. control group; # p<0.05 *vs*. H/R group; & p<0.05 *vs*. PC group; $ p<0.05 *vs*. H/R + NaHS group; § p<0.05 *vs*. PC + NaHS group.

**
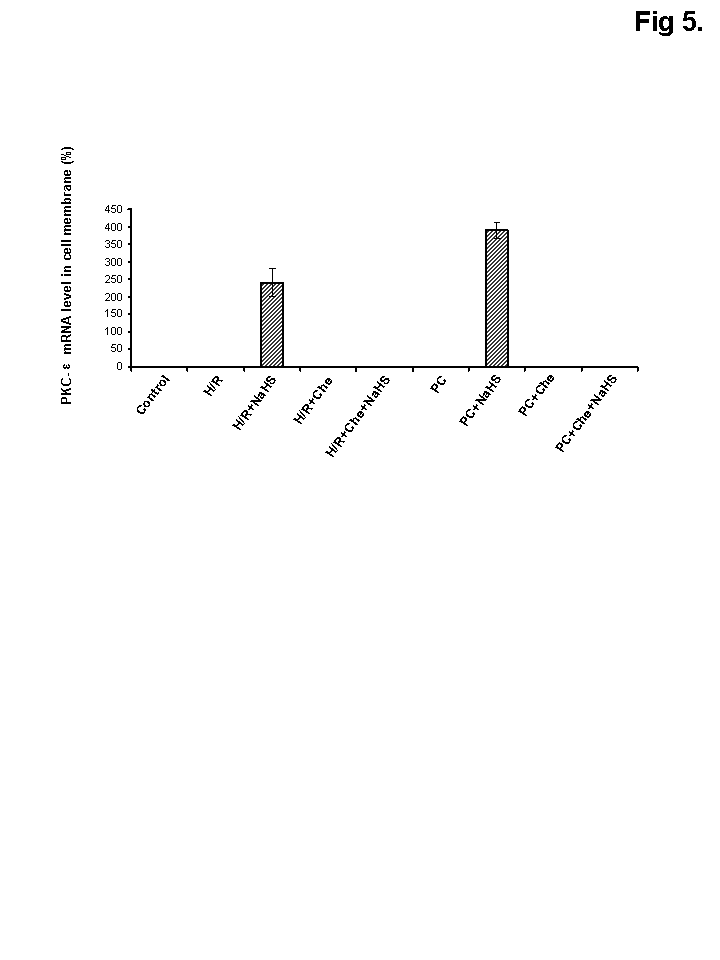
**

**Figure S5. The effect of exogenous H_2_S on PKC-ε** **mRNA levels in the cell membrane in the D-galactose age-induced cardiomyocytes.** The level of PKC-ε mRNA was tested using Real-Time PCR. The data were normalized to the GAPDH. All data were from four independent experiments. Control, H/R, H/R + Che, H/R + Che + NaHS, PC, PC + Che and PC + Che + NaHS groups showed no translocation of PKC-ε to cell membrane. H/R + NaHS and PC + NaHS groups showed translocation of PKC-ε to cell membrane.
